# Technical evaluation of Virtual Touch™ tissue quantification and elastography in benign and malignant breast tumors

**DOI:** 10.3892/etm.2014.1875

**Published:** 2014-08-04

**Authors:** QUAN JIANG, YUAN ZHANG, JIAN CHEN, YUN-XIAO ZHANG, ZHU HE

**Affiliations:** Department of Ultrasound, Pudong New Area People’s Hospital, Shanghai 201200, P.R. China

**Keywords:** breast tumors, ultrasound, elasticity imaging technology, Virtual Touch™ tissue quantification

## Abstract

The aim of this study was to investigate the diagnostic value of the Virtual Touch™ tissue quantification (VTQ) and elastosonography technologies in benign and malignant breast tumors. Routine preoperative ultrasound, elastosonography and VTQ examinations were performed on 86 patients with breast lesions. The elastosonography score and VTQ speed grouping of each lesion were measured and compared with the pathological findings. The difference in the elastosonography score between the benign and malignant breast tumors was statistically significant (P<0.05). The detection rate for an elastosonography score of 1–3 points in benign tumors was 68.09% and that for an elastosonography score of 4–5 points in malignant tumors was 82.05%. The difference in VTQ speed values between the benign and malignant tumors was also statistically significant (P<0.05). In addition, the diagnostic accuracy of conventional ultrasound, elastosonography, VTQ technology and the combined methods showed statistically significant differences (P<0.05). The use of the three technologies in combination significantly improved the diagnostic accuracy to 91.86%. In conclusion, the combination of conventional ultrasound, elastosonography and VTQ technology can significantly improve accuracy in the diagnosis of breast cancer.

## Introduction

The hardness of breast lesions is closely associated with the degree of malignancy, and is the basis for the evaluation of benign or malignant tumors by elastosonography. Elastosonography reflects the hardness of lesions by detecting the differences in hardness between various structures. Conventional elastography can be used to estimate breast tissue elasticity (1) but there are certain disadvantages with freehand compression: the elasticity map obtained is highly dependent on the compressibility limits under stress of the organ and on the extent of the tissue compression applied.

The diagnosis of breast lesions with elastosonography using a 5-point scoring system is relatively subjective (2), as the information displayed relates to a local strain estimated at a given location in the tissues, but this depends on the mechanical properties of the surrounding tissues and it is not quantitative. It has been shown that objective quantification of tissue elasticity could now be possible using acoustic radiation force impulse (ARFI) elastometry. With this system, instead of using external compression, commercially available ultrasound scanners are used to generate short-duration acoustic radiation forces that impart small (1–10 mm) localized displacements in the tissue. The response of the tissue to the radiation force is detected using conventional B-mode imaging pulses in order to track the displacement of tissue which correlates with the local stiffness of the tissue. As a result, two-dimensional images of tissue displacement are generated by the repetition of this process along multiple image lines (3,4). The generated waves can provide a qualitative response (gray-scale map) by virtual touch tissue imaging or a quantitative response (shear wave velocity values, measured in m/s) by virtual touch tissue quantification (VTQ), dependent upon the interactions with the transducer (5). ARFI has been evaluated in various tissues using both the grey-scale map and the shear wave speed (6).

In this study, 86 patients with breast lesions received routine ultrasound, elastosonography and VTQ examinations. The aim of the present study is to investigate the diagnostic value of elastosonography and VTQ technologies in the development of benign or malignant breast tumors.

## Materials and methods

### General information

Between July 2011 and March 2012, 86 females who had undergone surgical resections of breast lesions in Pudong New Area People’s Hospital (Shanghai, China) were selected for the study. The age of the patients was 18–72 years with a mean of 36.8±13.6 years. The lesion diameters ranged between 7 and 32 mm with an average of 16±8 mm. All lesions were confirmed by histopathology. This study was approved by the Ethics committee of Pudong New Area People’s Hospital (Shanghai, China) and patient informed consent was obtained prior to the study.

### Instruments and methods

The Acuson S2000™ Ultrasound System with color Doppler imaging (Siemens Healthcare, Erlangen, Germany) was used for the elastosonography and VTQ technologies. A 9L4 linear array probe was used with a frequency of 9 MHz. A routine ultrasound examination was initially performed to observe the gray-scale sonographic characteristics of the lesions, including shape, size, edge, border, internal and posterior echo, and color Doppler flow characteristics. The elastosonography function was then started to determine the region of interest (ROI). The sampling frame used was ideally greater than the scope of the lesion and the quality control indicators were set between 60 and 80. The image with the highest quality was selected for the elasticity score. The elastosonography images were color-coded to represent the elastic strain of the tissues. From soft to hard, the hardness was coded as purple, blue, green, yellow and red. Green represented the average hardness of tissue elasticity in the ultrasound sampling frame. Red and yellow indicated that the hardness was greater than the average hardness; purple and blue indicated that the hardness was less than the average hardness. VTQ inspection was performed following elastosonography. If the lesion was larger than the sampling frame, the sampling frame was placed in the center of the lesion. If the lesion was smaller than the sampling frame, the lesions were located in the center of sampling frame. The VTQ function was started, following which the machine automatically gave the measured VTQ speed values by the ROI. Each lesion was measured five times and the mean value was selected.

### Diagnostic criteria

The conventional ultrasound diagnostic criteria for malignant lesions comprised an irregular shape and burr-like edge, internal microcalcifications, rear attenuation, internal rich blood flow and a resistive index (RI) >0.75. The diagnostic criteria for benign lesions included clear boundaries, insufficient blood flow and an RI ≤0.75.

The flexible ultrasound diagnostic criteria were based on the literature (7,8), whereby the elasticity image was scored according to the different colors in the lesions, as follows: 1 point, purple, green and red; 2 points, the lesion and surrounding tissue were green; 3 points, red, green and yellow lesions, with >50% green; 4 points, red, yellow and white lesions, with 50–90% red; 5 points, >90% of the lesion area was red. Elastosonography scores of 1–3 points were diagnosed as benign lesions and 4–5 points as malignant lesions.

The pathological findings were utilized as the gold standard. The sensitivity was taken as the vertical axis and one minus the specificity as the horizontal axis. The receiver operating characteristic curve was then created and the threshold was analyzed. With regard to the diagnostic criteria for the three techniques combined, lesions with at least two positive results from the three techniques were defined as malignant, the rest as benign.

### Statistical analysis

Statistical Analysis System (SAS) 8.0 statistical software (SAS Institute, Inc., Cary, NC, USA) was used for the statistical analysis. The detections rates for the benign and malignant elastosonography scores and the VTQ speeds in each group were compared using a χ^2^ test. P<0.05 was considered to indicate a statistically significant difference. Taking pathological findings as the gold standard, the diagnostic performance using a combination of the three methods of routine ultrasound, elastosonography and VTQ technology was calculated.

## Results

### Pathology results

Benign breast lesions were histologically confirmed in 47 out of 86 patients; out of these 47 patients, fibroadenoma and adenosis were diagnosed in 38 and nine patients, respectively. Malignant tumors were found in 39 patients; 35 patients had invasive ductal carcinoma, one patient had invasive lobular carcinoma, two patients had medullary carcinoma and one patient had ductal carcinoma *in situ*.

### Benign and malignant elastosonography scores

**Figure 1:**
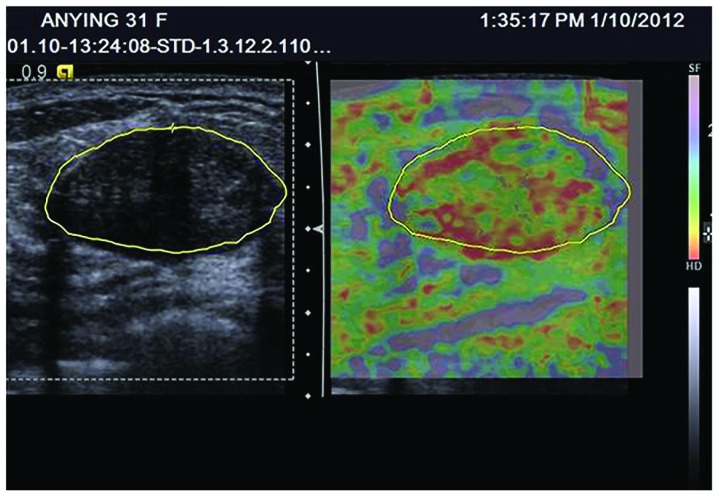
Surgical pathology in a 31-year-old woman with a palpable mass in the inner upper quadrant of her left breast proved an adenofibroma. Left image: Two dimensional sonogram. Right image: Elastography map, 3 points.

**Figure 2:**
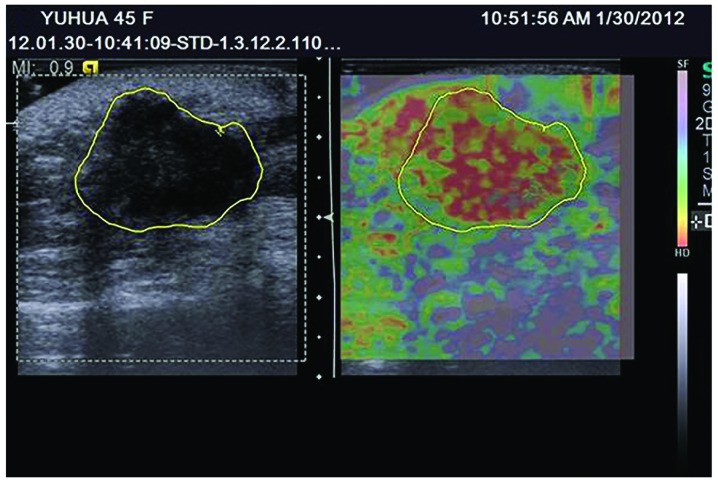
Surgical pathology in a 45-year-old woman with a palpable mass in the outer upper quadrant of her right breast proved an invasive ductal carcinoma of the breast. Left image: Two dimensional sonogram. Right image: Elastography map, 5 points.

The elastosonography scores are shown in Table I. The detection rate of benign breast lesions with an elastosonography score of 1–3 points was 68.09%, which was significantly higher than that of malignant tumors (P<0.05). The benign breast lesion elastosonography scores were mainly 1–3 (Fig. 1). The detection rate of malignant tumors with an elastosonography score of 4–5 points was 82.05%, which was significantly higher than that of benign tumors (P<0.05), and the malignant breast lesion elastosonography scores were mainly 4–5 (Fig. 2). The detection rates of benign and malignant tumors with an elastosonography score of 4 points were 21.28 and 20.51%, respectively; this difference was not statistically significant (P>0.05).

### VTQ speed values in benign and malignant tumors

**Figure 3:**
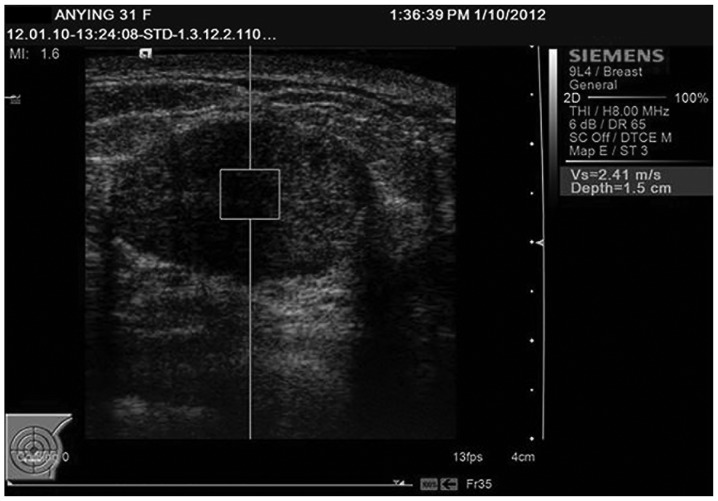
Surgical pathology in a 31-year-old woman with a palpable mass in the outer upper quadrant of her right breast proved an adenofibroma. VTQ detection map (VTQ value, 2.41 m/sec). VTQ, Virtual Touch™ tissue quantification.

**Figure 4:**
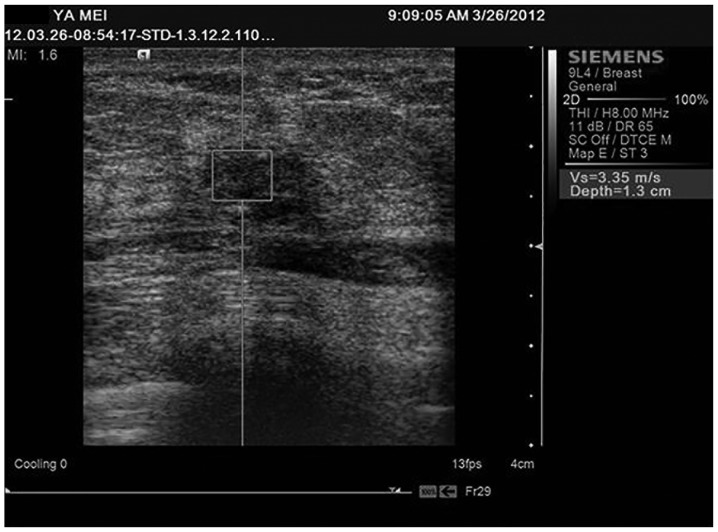
Surgical pathology in a 48-year-old woman with a palpable mass in the inner upper quadrant of her right breast proved an invasive ductal carcinoma detection map (VTQ value, 3.35 m/sec). VTQ, Virtual Touch™ tissue quantification.

The VTQ speed results are presented in Table II. The detection rate of benign breast tumors with a VTQ speed value of <2.98 m/sec was 74.47%, which was significantly higher than that of the malignant tumors (P<0.05); the VTQ speed of benign breast tumors was mainly <2.98 m/sec (Fig. 3). The detection rate of malignant breast tumors with a VTQ speed value of ≥2.98 m/sec was 79.49%, which was significantly higher than that of the benign tumors (P<0.05); the VTQ speed of malignant breast tumors was mainly ≥2.98 m/sec (Fig. 4).

### Performance of the four types of inspection methods in the diagnosis of benign and malignant tumors

The diagnostic performance results are presented in Table III. Statistically significant differences were observed between the diagnostic accuracy of conventional ultrasound, elastosonography, VTQ technology and the three methods combined (P<0.05).

## Discussion

To date, there has been a constant pursuit of the ultrasound characteristics of non-benign breast lesions, otherwise referred to as malignant tumors. Two-dimensional gray-scale ultrasound, color Doppler and pulsed Doppler techniques were gradually developed to distinguish between benign and malignant breast lesions. However, both in the two-dimensional gray-scale images and in the images obtained using color Doppler flow imaging, a large degree of overlap existed between benign and malignant lesions, rendering it difficult for conventional ultrasound make an accurate diagnosis. As a result, the emerging elastosonography and VTQ technologies were applied to the study of breast disease (9–11). Elastosonography took the biological tissue elasticity (or stiffness) and the biological characteristics of lesions as the theoretical basis, and provided a novel approach for the differential diagnosis of breast lesions. The principle underlying VTQ is that when the ROI tissues are affected by the pulse wave, shear waves accompanied by lateral transfer movement are produced; the probe pulse sequence then collects these subtle changes and the system records and calculates its speed. This speed is equivalent to or representative of tissue elasticity (12). VTQ is the absolute tissue quantitative indicator, and can not only be contrasted with neighboring tissues, but also with tissue images from different patients. VTQ compensates for the defects of the qualitative or semi-quantitative evaluation of previous elastosonography methods.

The results of this study showed that the elastosonography scores of the benign breast tumors were mainly 1–3 points, with a detection rate of 68.09%. The elastosonography scores of the malignant breast tumors were mainly 4–5 points, with a detection rate of 82.05%. The difference in the detection rate of the elastosonography score between the benign and malignant tumors was statistically significant (P<0.05). Elastosonography imaging reflected the overall hardness level of the tumor tissue compared with that of the surrounding normal breast tissue. The assessment of VTQ technology showed that the benign breast tumor VTQ speeds were mainly <2.98 m/sec, with a detection rate of 74.47%. The VTQ values of the malignant breast tumors were mainly ≥2.98 m/sec, with a detection rate of 79.49%. With regard to the VTQ speed values, the detection rates of the benign and malignant breast tumors showed a significant difference (P<0.05). The VTQ speed values increased in parallel with increasing tissue hardness. These findings may be explained by the fact that, in most cases, the benign lesions had a soft texture whereas the malignant lesions had a hard texture. The elastosonography scores were closely associated with the pathological changes, but mainly reflected the elasticity characteristics of the lesions. In 1998, Krouskop *et al* (13) described the elasticity of different breast tissues in decreasing order: Invasive ductal carcinoma, non-invasive ductal carcinoma, breast fibrous tissue, normal breast tissue and adipose tissue. It was speculated that the larger the tissue elasticity coefficient, the greater the hardness. The study by Konofagou (14) showed that the malignant lesions had more complex and irregular boundaries, therefore exhibiting poor mobility, reduced relative strain and a large elasticity coefficient.

The results of this present study showed that the benign and malignant tumors had a certain overlap in hardness. Although in most cases, the malignant breast tumors had a harder texture than the benign tumors, this was not absolute. In certain histological types, including medullary carcinoma of the breast, the pathology findings showed that the internal medullary carcinoma tumor cells were in a follicular arrangement and contained a lower fiber composition (15), therefore leading to a relatively soft texture. In this study, two cases of medullary carcinoma had elastosonography scores of 2 and 3 points, and the VTQ velocity values were 2.06 and 2.23 m/sec, indicating the soft texture of medullary carcinoma. Certain fibroadenomas with high degrees of fibrosis exhibited a relatively high hardness. At the same time, a variety of pathological changes within the breast lesions may also have an impact on the hardness of the lesions. The hardness may increase in benign lesions when fibrosis or calcification occur and the hardness may decrease in malignant lesions when necrosis or hemorrhage occur.

This study showed that the sensitivity, specificity and accuracy of elastosonography in the diagnosis of breast lesions were 84.62, 63.83 and 73.26%, respectively. The sensitivity, specificity and accuracy of VTQ technology in breast lesions were 76.92, 76.60 and 76.74%, respectively. When elastosonography and VTQ technology are used separately, they can only provide information regarding lesions and tissue elasticity. The hardness of benign and malignant tumors may overlap which would result in misdiagnosis. In this study, the combined diagnosis using three types of technology significantly improved the diagnostic accuracy to ≤91.86%.

In conclusion, elastosonographies of breast lesions and VTQ technology can indirectly and directly, respectively, reflect the hardness of breast lesions. This can contribute to the identification of benign and malignant tumors. However, due to the overlap in the hardness of benign and malignant tumors, actual clinical application would benefit from combining these methods with conventional ultrasound to optimize the accuracy of breast tumor diagnoses.

## Figures and Tables

**Table I:** Elastography scores of benign and malignant breast lesions.

		Ultrasound elastography score					
		
		1–3		4		4–5	
				
Pathology	Number of cases (n)	n	Detection rate (%)	n	Detection rate (%)	n	Detection rate (%)
Benign	47	32	68.09	10	21.28	15	31.91
Malignant	39	7	17.95	8	20.51	32	82.05

**Table II:** VTQ speed values of benign and malignant breast lesions.

		VTQ speed value (m/sec)			
		
		V<2.98		V≥2.98	
			
Pathology	Number of cases (n)	n	Detection rate (%)	n	Detection rate (%)
Benign	47	35	74.47	12	25.53
Malignant	39	8	20.51	31	79.49

VTQ, Virtual Touch™ tissue quantification; V, speed value.

**Table III:** Comparison of the diagnostic performance of four types of inspection methods for benign and malignant breast lesions.

Inspection method	Preoperative diagnosis		Postoperative pathology				Sensitivity (%)	Specificity (%)	Accuracy (%)

			Malignant (n=39)		Benign (n=47)				
		
	Malignant (n)	Benign (n)	Correct diagnosis	Misdiagnosis	Correct diagnosis	Misdiagnosis			
Conventional ultrasound	41	45	31	8	37	10	79.49	78.72	79.07
Elastosonography	50	36	33	6	30	17	84.62	63.83	73.26
VTQ	41	45	30	9	36	11	76.92	76.60	76.74
Combined diagnosis	40	46	36	3	43	4	92.31	91.49	91.86

VTQ, Virtual Touch™ tissue quantification.
